# The effects of vitamin C and menthone on acyclovir induced DNA damage in rat spermatozoa: An experimental study

**Published:** 2018-11

**Authors:** Shima Toghiani, Nasim Hayati Roudbari, Gholam Reza Dashti, Shahla Rouzbehani

**Affiliations:** 1 *Department of Biology, Science and Research Branch, Islamic Azad University, Tehran, Iran.*; 2 *Department of Anatomical Sciences, School of Medicine, Isfahan University of Medical Sciences, Isfahan, Iran.*; 3 *Department of Biology, Falavarjan Branch, Islamic Azad University, Isfahan, Iran.*

**Keywords:** Vitamin C, Menthone, Acyclovir, DNA damage, Rat

## Abstract

**Background:**

Acyclovir (ACV) is known to be toxic to gonads, inducing apoptosis in the reproductive system. The beneficial effects of vitamin C (Vit C) and menthone, both as antioxidant agents on various organs has been reported.

**Objective:**

This study evaluated the potential role of the Vit C and menthone on the DNA damage in rat spermatozoa induced by the ACV.

**Materials and Methods:**

In this experimental study, adult male albino Wistar rats with average weight of 250±10 gr, were divided into six groups (n=18/each), as: ACV (15 mg/kg/day), ACV+Vit C (20 mg/kg/day), ACV+ menthone (100 µl/d), ACV+ menthone (250 µl/d), ACV+ menthone (400 µl/day) and control group without any treatment. At the end of experiment, the animals were sacrificed and sperm samples were collected and isolated in phosphate-buffered saline and examined by TUNEL staining process. The percentage of TUNEL positive spermatozoa was evaluated by fluorescence microscopy. Each experiment was performed in three repeats.

**Results:**

Male rats exposed to ACV had significant increase in DNA damages in comparison to other groups. The percentage of TUNEL positive sperm cells was 90.83 (p<0.001) in ACV group. The protective role of both antioxidants used in high dose, compensate the adverse effects of the ACV. The results showed that the percentage of apoptotic sperm in the ACV+Vit C group was 16.38 (p<0.001) and in the ACV+ menthone (400 µl/d) group was 16.05 (p<0.001).

**Conclusion:**

The present results showed that Vit C and menthone at higher dose have a good compensatory effect with significant reduction in DNA damages in sperm cells by reversing the adverse effect of ACV on the reproductive system in male rat.

## Introduction

The development of germ cells is controlled by various factors, such as follicule stimulating hormone and testosterone hormones. A number of germ cells, before reaching maturity, dies and undergo physiological apoptosis )^1^). Environmental factors such as irradiation, chemotherapy or antiviral drugs result in DNA damages in cells, causing germ cells apoptosis and spermatogenesis cytotoxicity (2, 3). In reproductive system, genetic damage is transferred from one generation to another (4). Thus, it is important to investigate the testicular apoptosis and genotoxic effects of agents along with protective role of antioxidants on germinal cells. 

The side effects of these drugs have not been thoroughly studied in relation to spermato-toxic effects. Acyclovir (ACV) can cause micronuclei formation in some somatic cells and is capable to damage cellular DNA in the non-infected cells (5). It has potential effect on chromosome breaks in vitro and in vivo (6). The male gonad of reproductive system is very susceptible to damage when exposed to toxic effect of chemotherapy and drugs (7, 8). There are sparse and inclusive studies on the influence of ACV on reproductive performances in male gonads of different species. The present research wants to investigate the idea that whether ACV can cause potential adverse effect on gonads of male rats and which combinations of antioxidants, may prevent its harmful effects?

Recently, many traditional medicines are being widely used for prevention and treatment of different human diseases like cancer, reproductive infections, cardiovascular diseases, bacterial, and viral infections (9-12). The herb of Mentha piperita (M. piperita) was reported by several studies to have antioxidant, cytotoxic, antiallergenic, antiviral and antibacterial activities (13). The oil of M. piperita (meo) has antimicrobial and antioxidant activities (14). The chemical composition of M. piperita (MEO) is mainly composed of menthol, menthone and menthe acetate. Menthone (C10H18O) comprised 20.7-28.8% of essential oil of Menthe genus (15). The widespread use of M. piperita in traditional medicines has inspired us to investigate the effect of menthone, in rat spermatogenesis. 

Vitamin C (ascorbate) is being investigated in clinical trials as the first line of antioxidant defense against immune system deficiencies and cardiovascular diseases (16). Vit. C has an effect of potent water soluble antioxidant property that scavenges reactive oxygen and nitogen species by preventing oxidative damage to important biological macromolecules. Vit C has a very strong supportive, protective effect along with impressive anti-apoptotic power. The expression level of caspase 3 in Vit C receiving group was found to be very effective (17).

Interestingly, to our knowledge no previous experimental studies have suggested menthone as a potent supportive agent of spermatogenesis. The aim of this study was to evaluate the antioxidant effect of Vit C and various concentration of menthone on DNA damage induced by ACV in the spermatozoa of adult male rats.

## Materials and methods

**Animals and** **drug treatment**

In this experimental study, adult male albino Wistar rats (250±10 gr), were divided randomly into six groups (n=18/each) purchased from Pasture Institute of Iran. All animals were treated in accordance to the Principles of Laboratory Animal Care. This study was carried out in Isfahan University of Medical Sciences. The animals were kept under specific hygienic conditions on a constant 12 hr light/dark cycle with a temperature ranged 25±2^o^C and mean relative humidity of 50±5% in standard separate cages. 

Animals were allowed to acclimatize for one week before experimental study. They were fed standard pellet food diet and had access to water ad libitum. ACV (MYLAN Company, France) was dissolved in distilled water before injection. One group served as control group receiving only water and food. Group two was administered dose of 15 mg/kg/d ACV. The other groups of rats was administered with supportive treatment as followed ACV+Vit C 10 mg/day, ACV+menthone 100 µl/d (M1), ACV+menthone 250 µl/d (M2) and ACV+menthone 400 µl/d (M3) for 48 days (17). (menthone purchased from MERCK (catalog number: 8.41059.0025, Germany). At the end of experiment, the animals were anesthetized with 10% ketamine solution and sacrificed with an interval of 24 hr after the last treatment. 

**Sampling and isolation of spermatozoa:**

**Sperm count**

Epididymal spermatozoa were collected by cutting the caudal epididymis into segments of approximately 1 mm and transferred into 1 ml of phosphate buffered solution (pH=7.2). Spermatozoa from caudal regions were completely removed by vortexing gently in phosphate-buffered saline (PBS), and the tissue debris was allowed to settle for 5 min. Spermatozoa suspension was incubated for 5 min in 37^o^C with 5% of CO_2_ to let the sperm swim out of the epididymal tubules. A few drops of diluted sperm suspension was placed into a hemocytometry (Neubauer counting chamber) with a depth of 0.1 mm, and allowed to stand for 5 min. The sperm heads were counted and expressed as million/ml of suspension (18-20). A minimum of 100 sperms were observed for motility and percentage of motile sperms was recorded for each animal.

**Determining the percentage of motile sperms**

One drop of sperm suspension was placed on a glass slide, covered with a coverslip, and the percentage of sperm motility with progressive movement was estimated under a light microscope (Olympus Co., Tokyo, Japan) with a magnification of ×10, then with ×40 in 10 fields and the percentage of sperms with motile and nonmotile sperm were calculated. Motility was expressed as the percentage of motile sperm to the total number of sperm (19-21).

**Morphological analysis of sperms**

The normal sperm morphology was assessed by papanicolaou (PAP) staining. The sperms in terms of normal and abnormal shape of the head and tail were analyzed in prepared slides. The smears were fixed by ethanol-ether (1:1) for 5 min and then stained with PAP staining solutions according to world health organization guidelines. The sperms presenting the defects in shape and structure of either head or tail were considered as abnormal and the mean data were recorded as percentage incidence of total abnormalities (22, 23)

**Sperm DNA fragmentation detection by fluorescence microscopy:**

Sperm DNA fragmentation was evaluated with terminal deoxynucleotidyl transferase mediated-deoxyuridine tri phosphate nick and labeling assay (TUNEL method). The DeadEnd™ Fluorometric TUNEL System was performed for specific detection and quantification of apoptotic cells within a cell population (Apoptosis Detection System Fluorescein; Promega, Mannheim, Germany, G3250). Sperm suspension was centrifuged for 5 min at 300 g. Supernatant was discarded and the Pellet was washed in PBS (pH=7.4). 

One drop of sperm suspension was placed onto slide, air-dried and fixed by immersing in 4% methanol-free formaldehyde in PBS for 25 min at 4^o^C. The sperm smear slides were permeabelized in Triton X-100 for 5 min, rinsed and subsequently equilibrated with equilibration buffer for 5 to 10 min at room temperature. Nucleotide mix and rTdT were prepared for test and control slides of specimens. Nuclear DNA strand breaks was labeled with fluorescein-12-dUTP and kept for 60 min at 37^o^C in a humidified chamber to protect it from direct light. The reactions were stopped by immersing the slides in 2X SSC for 15 min at room temperature. The slides were washed three times for 5 min in PBS solution to remove unincorporated fluorescein-12-dUTP. 

The slides were stained with freshly diluted propidium iodide solution (1 μg/ml in PBS) for 15 min at room temperature in the dark. The slides were washed again three times for 5 min by immersing in PBS. The fluorescein-12-dUTP-labeled DNA was analyzed with a fluorescence microscope (Nikon Eclipse E400) with appropriate filters (460-470 nm). 200 nuclei of sperm cells were evaluated on each slide. The sperm with DNA damage were considered TUNEL positive and recorded (23). Apoptotic sperms look like golden due to the combination of antibody green fluorescence and red fluorcentric color propodeum iodide background. The nuclei of sperm cells with fragmented DNA showed yellow color, whereas nuclei of normal cells were seen as red.

**Ethical consideration**

This experimental study was conducted in accordance with the internationally accepted principles of laboratory animals use and care research with institutional guidelines of ethical committee (code no: 10795.).

**Statistical analysis**

Results for each group are expressed as mean±S.D. and comparison of data between groups for significance by ANOVA and Least Significant Difference (LSD) post hoc test were used. For statistical analysis, we used statistical package for social sciences, (SPSS, version 19.0 SPSS Inc. Chicago, Illinois, USA). The p<0.001 was considered statistically significant.

## Results

In this experimental study, the descriptive results of sperm count in the control, ACV, ACV/Vit C, ACV/M1, ACV/M2, ACV/M3 were analyzed. Comparison of the sperm count in all the groups except ACV showed significant increase. In addition, we observed a significant increase in the sperm count of groups receiving Vit C and high dose of menthone when compared with ACV group (table I). 

The percentage of motility in the control, ACV, ACV/Vit C, ACV/M1, ACV/M2, ACV/M3 were evaluated respectively (p<0.001). The motility of the sperms in the groups receiving Vit C, high dose of M and normal group, when compared was found to be similar. We observed a significant decrease in the sperm motility in ACV group compared with control group (table I). The percentage of abnormal morphology assessed by PAP staining in the control, ACV, ACV/Vit C, ACV/M1, ACV/M2, ACV/M3 were evaluated. The group of animals receiving ACV alone showed a significant increase in the percentage of abnormal sperms as compared to other groups. Comparison of percentage of normal sperm morphology showed a significant increase in the group following Vit C and high dose of menthone with ACV when compared with ACV group alone. In addition we observed a significant increase in the percentage of normal sperm morphology in ACV/M3 compared to ACV/M2 and ACV/M1 groups (Table I).

**Figure 1:**
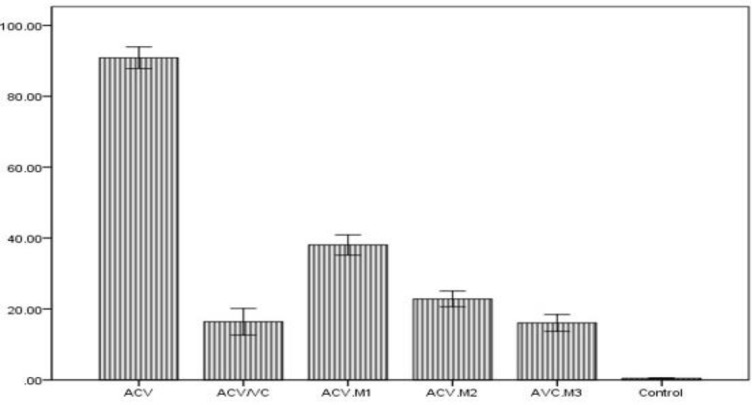
Effect of ACV, Vit C and different dosages of menthone on spermatozoa of adult male rats by TUNEL assay system in comparison to control group

**Figure 2:**
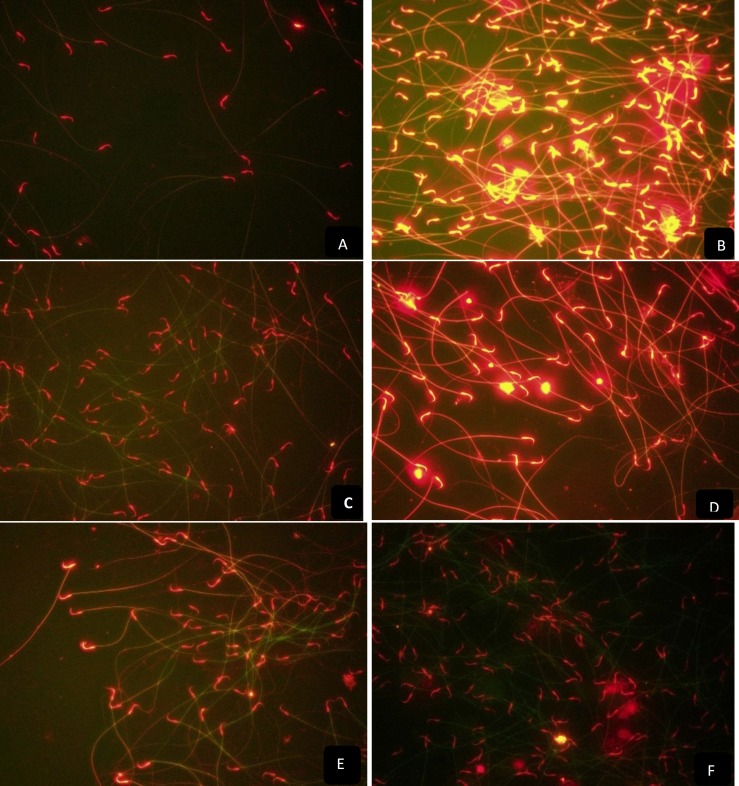
TUNEL assay of spermatozoa in six experimental groups. Positive apoptosis cells is brilliant golden yellow color and negative apoptosis cells is red color. A: spermatozoa smear of control rats shows normal TUNEL negative chromatin structure. B: smear of ACV treated rats show apoptosis in a grade range (90.84%), C: Vit C co-treated groups shows significantly reduced percentage of apoptotic spermatozoa (16.4%), D,E,F: and in different dosages of menthone groups, the percentage of TUNEL positive sperm cells were 38.05% for M1, 22.84% for M2 and 16.05% for M3 respectively. (400x, magnification).

The mean percentage of DNA damage assessed by TUNEL technique in the control, ACV, ACV/Vit C, ACV/M1, ACV/M2, ACV/M3 were 0.44, 90.83, 16.38, 38.0556, 22.8333 and 2.36322 respectively (Table II). The percentage of DNA damage showed significant decrease in the Vit C and Menthone group when compared with ACV group (Figure 1, 2). The antioxidants was found to have significant compensatory effect with an acceptable potent for protecting the sperm. (Table II, Figure 1, 2).

**Table I:** Effect of ACV, Vit C, and different dosages of Menthone on sperm parameters in adult male rats

**Group**	**Sperm count**	**Motile sperms (%)**	**Abnormal sperms (%)**
ACV	1.14×10^8^	28.5	14.9
ACV/Vit C	2.8×10^8^	84.61	0.8
ACV/M1	1.9×10^8^	59.05	4.19
ACV/M2	2.6×10^8^	73.33	1.6
ACV/M3	2.8×10^8^	84.11	0.7
Control	3.24×10^8^	100	0

ACV: Acyclovir

Vit C: Vitamin C

M1: Menthone (100µl)

M2: Menthone (250µl)

M3: Menthone (400µl)

**Table II:** Effect of ACV, Vit C, and different doses of Menthone, on sperm DNA damage assessed by TUNEL staining (p<0.001) (n=18/each)

**Group**	**Mean (%)**	**Std. error**
ACV	90.8333	3.03708
ACV/Vit C	16.3889	3.74614
ACV/M1	38.0556	2.86342
ACV/M2	22.8333	2.20701
ACV/M3	16.0556	2.36322
Control	0.4444	0.12052

ACV: Acyclovir

Vit C: Vitamin C

M1: Menthone (100µl)

M2: Menthone (250µl)

M3: Menthone (400µl)

## Discussion

Management of male infertility depends mainly on understanding the cellular and molecular aspects of spermatozoa. Now a day, much attention is being paid to the role of apoptosis of germinal cells which plays a critical role in reproduction. The present study was planned to estimate the potential role of antioxidants like Vit C and different dosages of menthone on spermatozoa impaired by acyclovir. The results showed that ACV has an adverse effect on spermatogenesis parameters; decreasing sperm concentration, progressive motility and increases abnormal sperm morphology. Menthone in a dose dependent manner and Vit C both could reduce the deleterious effects of acyclovir on spermatozoa. In this study, the highest dose of menthone and dose of Vit C showed to have good positive effect in supporting the spermatogenesis and thereby we observed a significant increase in the percentage of normal sperm morphology by PAP staining.

To our knowledge, there is no research study indicating the compensatory role of menthone on sperm parameters and DNA integrity. The Vit. C and menthone in higher doses showed to have a good compensatory effect by reversing the adverse effect of ACV ,thereby reducing the apoptotic sperm cells. The percentage of apoptotic cells in these two treatments groups was lower as compared to other experimental groups. It is known that apoptosis of germinal cells number plays a critical role in male infertility (24, 25).( Any damage in the germ cells, leads to changes in the function which can alter sperm count and motility. The agents that interfere with mitotic division show the efficacy to reduce the sperm count (25). 

Previous studies reported that ACV has an efficacy to damage cellular DNA in non-infected cells (25, 26). Several studies have reported that antiviral drugs such as Ribavirin and Gancyclovir decreases sperm count (27, 28) and acyclovir can increase the sperm abnormality and decrease the sperm count (20, 29). In the present study, our treatment was 48 days and the length of spermatogenesis in rat is approximately 39-45 days. Our findings were similar to the above experimental studies that reported ACV causes significant increase in the percentage of TUNEL positive sperms thereby impairing sperm morphology and motility. This study found a significant decrease in the number of spermatozoa as well as a decrease in the motility and the number of normal sperms.

In this study, we aimed to evaluate the effect of two antioxidant compounds on spermatogenesis. Menthone is a compound extracted from plants and ascorbic acid which has strong antioxidant properties (30, 31). Previous studies have shown that in Vit C deficient rats, extensive degeneration of spermatocytes was observed. So it is expected that treatment with an antioxidant such as Vit C can compensate the damages of oxidative stress and apoptosis induction by ACV. Many studies reported that Vit C has a significant role against oxidative stresses that induce apoptosis in the spermatogonial cells and it can decrease the genetic abnormalities in the male and female germ cells and has a significant protective role against the irradiation in in-vitro systems (32). Our findings were similar to the study by Farias and colleagues. Our study showed that Vit C increases the rate of spermatozoa, sperm count, morphology and decreases apoptosis in spermatogonial cells by preventing DNA fragmentation.

On the other hand, Menthone is one of the antioxidant compounds in the Mentha oil that have an antioxidant, and antibiotic effects (33, 34). This compounds have a significant effect in decreasing the expression of inflammation factors such as PEG2 and LTB4 in cycloxygenase and lipoxygenase pathways (34). Previous studies have demonstrated that Mentha oil and its compounds have high potential power in the throw down of the free radicals and hydrogen donors. Researchers found that antioxidant ability of Mentha oil in treated rats has a good improvement as compared to the control group (33). High dose level of menthone was reported to reflect good potential effect against changes induced by ACV by reducing the caspase 3 expression in sperm cells (19). There are rarely any studies on the influence of ACV along with Vit C and different doses of menthone as a co-treatment on reproductive potential of male fertility in spermatozoa of different species. To the best of our knowledge, this study is one of the first reports of in-vivo experiment that investigated the antioxidant role of menthone on the cytotoxic effect of acyclovir induced on the rat spermatozoa. 

It is now well known that beside semen parameters, sperm DNA damage and its etiologies can affect male fertility (35). In this study, the assessment of TUNEL assay showed that group treated with high dose of menthone, demonstrated to have a good protective effect on sperm DNA with decrease in apoptosis in comparison to the ACV treated group. Although the present study was the first study to evaluate the effect of in-vivo administration of menthone on sperm in animals by reducing the percentage of apoptotic cells remarkably and improved the sperm parameters. Vit C also showed a similar significant protective activity and thereby enhances survival of the sperm cells as seen in the group treated with high dose of menthone. According to the results of present study, we hypothesized and suggest the use of menthone in preventing the possible harmful effects of cytotoxic agents in reproduction.

## Conclusion

All the adverse effects of ACV are reversible by co-treatment with Vit C and M. The assessment of the anti-apoptotic effect of in-vivo administration of dose-dependent manner of menthone against ACV showed a good potential ability, reliability and validity against sperm DNA fragmentation and decrease in the cytotoxicity of the sperm cells against apoptosis. Further investigations are needed in preclinical research and clinical trials in order to illuminate the mechanism of action of menthone for its therapeutic effect and thereby to find out a feasible place in prevention or treatment of male infertility. 
